# A case-control study of Burkitt lymphoma in East Africa: are local health facilities an appropriate source of representative controls?

**DOI:** 10.1186/1750-9378-7-5

**Published:** 2012-03-13

**Authors:** Sonya Baik, Mike Mbaziira, Makeda Williams, Martin D Ogwang, Tobias Kinyera, Benjamin Emmanuel, John L Ziegler, Steven J Reynolds, Sam M Mbulaiteye

**Affiliations:** 1 Global Health Sciences, UCSF, San Francisco, CA, USA; 2 EMBLEM Study Office, St. Mary's Hospital Lacor, Gulu, Uganda; 3 Center for Global Health, National Cancer Institute, Bethesda, MD, USA; 4 Department of Surgery, St. Mary's Hospital Lacor, Gulu, Uganda; 5 Infections and Immunoepidemiology Branch, National Cancer Institute, Bethesda, MD, USA; 6 Division of Intramural Research, National Institute of Allergy and Infectious Diseases, National Institutes of Health, Bethesda, MD, USA; 7 Infections and Immunoepidemiology Branch, Division of Cancer Epidemiology and Genetics, National Cancer Institute, National Institutes of Health, Department of Health and Human Services, 6120 Executive Blvd, Executive Plaza South, Rm. 7080, MSC 7248, Rockville, MD 20852, USA

## Abstract

**Background:**

We investigated the feasibility and appropriateness of enrolling controls for Burkitt lymphoma (BL) from local health facilities in two regions in Uganda.

**Methods:**

BL case data were compiled from two local hospitals with capacity to diagnose and treat BL in North-west and North-central regions of Uganda during 1997 to 2009. Local health facility data were compiled from children attending four representative local health facilities in the two regions over a two week period in May/June 2010. Age and sex patterns of BL cases and children at local facilities were compared and contrasted using frequency tables.

**Results:**

There were 999 BL cases diagnosed in the study area (92% of all BL cases treated at the hospitals): 64% were from North-central and 36% from North-west region. The mean age of BL cases was 7.0 years (standard deviation [SD] 3.0). Boys were younger than girls (6.6 years versus 7.2 years, *P *= 0.004) and cases from North-central region were younger than cases from North-west region (6.8 years versus 7.3 years, *P *= 0.014). There were 1012 children recorded at the four local health facilities: 91% at facilities in North-central region and 9% from facilities in North-west region. Daily attendance varied between 1 to 75 children per day. The mean age of children at health facilities was 2.2 years (SD 2.8); it did not differ by sex. Children at North-central region facilities were younger than children at North-west region facilities (1.8 years versus 6.6 years, *P *< 0.001).

**Conclusions:**

While many children attend local health facilities, confirming feasibility of obtaining controls, their mean age is much lower than BL cases. Health facilities may be suitable for obtaining young, but not older, controls.

## Background

Infection with Epstein-Barr virus (EBV) [1-3] and *Plasmodium falciparum *malaria [4-9] have been implicated in endemic Burkitt lymphoma (BL), a B cell non-Hodgkin lymphoma (NHL) described in African children by Denis Burkitt fifty years ago [10]. Significant associations between EBV and BL have been reported in case-control [7,8] and prospective [2] studies, and the EBV can be detected, as clonal episomes, integrated DNA, or early EBV Early RNA protein in (95%) of tumors [3]. The connection between malaria and BL is more tenuous, based on geographical co-distribution of both conditions at a population level [4-6]. However, correlation at a population level may not be true at the individual level (also called ecological fallacy). Recently, significant association at the individual level between anti-malaria antibodies and BL has been reported case-control studies [7-9]. However, interpretation of differences in antibody levels found in a case-control setting is not straightforward as differences could reflect changes occurring after, not before, onset of BL- also called reverse causation bias [11]. Thus, the cause and effect relationship between malaria and BL remains an important, albeit unanswered, scientific and public health question [12].

Because the risk of malaria is influenced by host genes [13], the link between malaria and BL may be investigated indirectly by evaluating the association between malaria-resistance genes and BL. This approach has the merit of being free from reverse causation bias, and genotypes can be measured reproducibly. Three small studies [14-16] attempted to use this approach some 40 years ago by measuring frequency polymorphisms in the ß-globin gene associated with the sickle cell trait (HBAS). Significantly or marginally reduced risk of BL was noted in two studies [15,16], but not in the third study [14]. Subgroup analysis of the third study focusing on another haemoglobin variant known as haemoglobin C (HBAC), which is also linked with malaria resistance, noted a decreased frequency of that variant in children with BL. Although our understanding of the role of genes in malaria resistance has increased vastly since these pioneering studies were conducted, and includes more than 50 implicated variants in 25 genes, no studies have investigated the role of these genes in BL.

We are conducting a prospective case-control study of BL in East Africa entitled "Epidemiology of Burkitt lymphoma in East African children and minors" (EMBLEM: http://emblem.cancer.gov) [17,18] to investigate the association between malaria-resistance genes and BL. The study is being conducted in rural areas adjacent to rivers or lakes in northern Uganda, Western Kenya and northern Tanzania, where environmental exposure to malaria is intense and continuous for 7-12 months per year [19]. Given the ubiquity of malaria exposure in the region, we assume that carriage (or not) of malaria-resistance genes will be one of the main factors influencing lifetime burden of malaria in children living in the study area. Another assumption is that children residing in the same geographical area will have comparable exposure to malaria. Hence, appropriate controls would have to be matched on age and geographical area of residence. In this paper, we investigated whether local health facilities might be an appropriate source of controls for BL cases by comparing and contrasting age and sex patterns of children at local facilities versus those of BL cases diagnosed at two participating hospitals in the study region in Uganda.

## Methods

### Ethics statement

Ethical approval to conduct the EMBLEM Study was obtained from the Uganda National Council of Science and Technology (Reference number: HS 816) and National Cancer Institute Special Studies Institutional Review Board (Reference number 10-C-N133).

### Study population: primary study base and strategies to improve ascertainment

Prior to implementing the EMBLEM study, we conducted an epidemiological study to define the primary study base [18,20] and a pathology study to investigate the quality of BL diagnosis at St. Mary's Hospital, Lacor [17]. The epidemiological study revealed that 90% of cases treated at the hospital resided within a radius of 100 miles of the hospital. Most BL cases presented with symptoms lasting 4-8 weeks of disease onset [18]. Based on these findings, we defined the primary study base of EMBLEM as children residing in a geographical area within a radius of 100 mile from participating hospitals for at least 4 months. Only 8 (~3%) of BL patients treated during 1994 to 1998 at the Uganda Cancer Institute [8], which is 350 km away in the capital city of Uganda, were from the EMBLEM study area. This suggests that BL cases tend to seek care at hospitals near their home. The pathology study at Lacor revealed that accuracy of pathology diagnosis although high (82%), it was variable (58% to 88%) [17]. Thus, the quality of pathology needed to be improved to obtain a complete and accurate BL case ascertainment from the primary study base.

To improve case ascertainment from the primary study base, EMBLEM has implemented four basic strategies: a) introduced free protocol-based BL treatment at participating hospitals to encourage case referral; b) introduced free diagnostic services at no cost to the patient; c) introduced culturally adapted BL *education and awareness messages *using illustrated posters http://emblem.cancer.gov/resources/index.html, animations, and audio, video, and radio messages targeting the community; and d) introduced community mobilization and training of local leaders and community research assistants to inform the community about BL, services, research, and improve follow up of cases. Together, these initiatives should improve case ascertainment in the study area by reducing time to presentation and boost case detection and referral to participating hospitals.

### Historical BL case distribution

BL data for children aged 0-15 years diagnosed during 1997 and 2009 were compiled from St. Mary's Hospital, Lacor, (Gulu) in North-central, and Kuluva Hospital (Arua) in North-west, two hospitals with capacity to diagnose and treat BL in the region. Age, sex, month and year of diagnosis and geographical area of residence (village, parish, sub-county, county, and district) for BL cases diagnosed at the two hospitals was abstracted and computerized [18] by research associates.

### Children at local health facilities

Data on children attending local health facilities were compiled over a 10-day period in May/June 2010 from four representative local health facilities in the EMBLEM study region. The facilities evaluated represent the lowest level local health facility in the country, called Health Centre II (HCII) in Uganda. The facilities serve a population residing in geographical area called a parish (comprising 3-6 villages or ~1000 to 7000 people). They provide basic outpatient services including immunization. There are no government run clinics at the village level because those facilities were replaced by an ambulatory village health worker team [21]. Facilities that serve higher level administrative areas, such as the sub-county (3-6 parishes are called HCIII facilities) or county (3-6 sub-counties, are called HCIV facilities) were considered, but deemed to be similar to hospitals and, therefore, unsuitable for EMBLEM needs. For each child, information on sex, age, village, parish, district, symptoms, reason for attending was compiled.

### Statistical comparisons

Distribution of BL by sex, tumor anatomic site (only face or head tumors, abdominal tumors with or without face or head involvement, and others sites, including unspecified), and age group (0-3, 4-6, 7-9, 10-12, and 13-15 years) were analyzed using frequency tables. Chi-squared tests were used to test associations between categorical variables and Student's *t *-tests to test the equality of means for continuous variables. Calendar year, geographical region, and age were considered to be important proxies of intrinsic and extrinsic exposures, so these patterns were examined graphically.

## Results

### Historical BL cases

**Figure 1:**
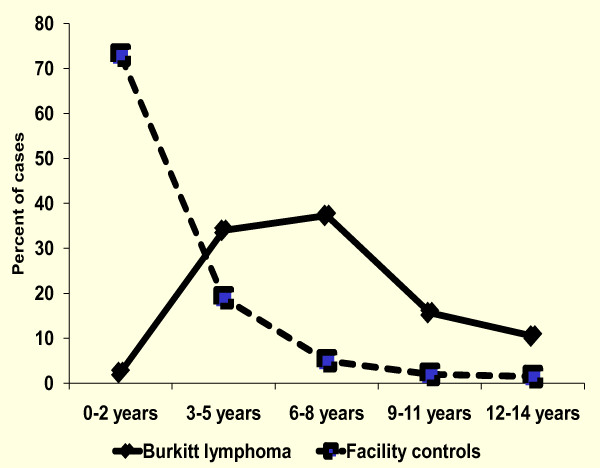
**Age distribution of historical Burkitt lymphoma cases (1997-2009) from two hospitals in northern Uganda vs. potential facility-based controls from four local health centres from the same region**.

There were 1088 BL cases diagnosed at the two hospitals; 999 (92%) were from the EMBLEM study region, including 64% from North-central and 36% from North-west region (Table 1). Most (62%) BL cases were male. The mean age of the cases was 7.0 years (standard deviation [SD], 3.0). The majority of cases were aged between 3 and 8 years; only 2% of the cases were aged 0 to 2 years (Figure 1). Slightly over a half of the cases presented with abdominal tumours, with or without face or head involvement; 37% presented with face or head tumours.

**Table 1:** Characteristics of Burkitt lymphoma cases from Northern Uganda during 1997-2009

	North-central region		North-west region		Total	
**Characteristic**	**n**	**%**	**n**	**%**	**N**	**%**

**Area of residence**						

Outside study area	59	9	30	8	89	8

Within study area	637	91	362	92	999	92

**Sex**						

Male	433	62	250	60	683	62

Female	263	38	164	40	427	38

**Age group, years**						

0 - 2	8	1	18	5	26	2

3 - 5	255	37	117	29	372	34

6 - 8	261	37	147	37	408	37

9 - 11	111	16	61	15	172	16

12 - 14	61	9	54	14	115	11

**Tumour anatomic site**						

Abdomen (± face)	365	52	198	48	563	51

Face only	238	34	167	40	405	36

Central nervous system	55	8	7	2	62	6

Others	18	3	24	6	42	4

Not recorded	20	3	18	4	38	3

Compared to BL cases from North-central region, cases from North-west were older (7.3 years versus 6.8 years, *P *= 0.014). In region-specific analyses, boys were younger than girls in cases from North-central region (6.6 years vs. 7.2 years, P = 0.004) and BL cases involving the face or head tumors were younger age than abdominal tumors (6.1 vs. 7.2 years, P < 0.001). By contrast, among the cases from North-central region, the mean age of BL cases was similar among boys and girls (7.3 years vs. 7.2 years, *P *= 0.79) and similar for cases involving face or head versus abdominal tumors (7.0 vs. 7.4 years, *P *= 0.28).

**Figure 2:**
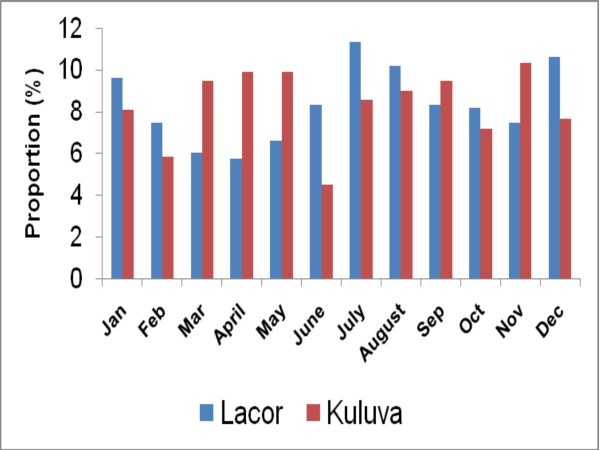
**Distribution of Burkitt lymphoma cases in northern Uganda by district and hospital where they were admitted (1997-2009)**.

**Figure 3:**
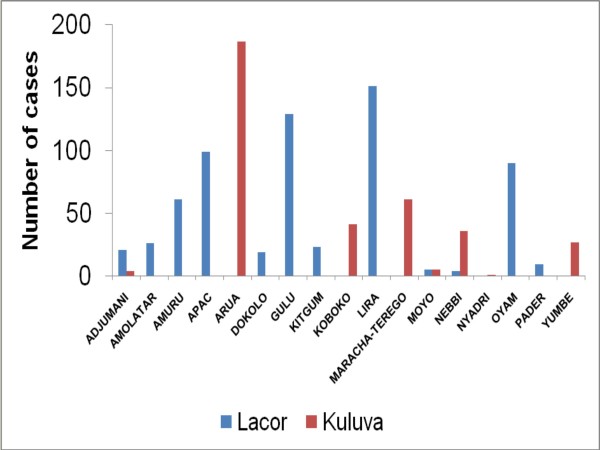
**Distribution of Burkitt lymphoma cases by calendar month and hospital (1997-2009)**.

The proportion of cases varied by calendar month (Figure 2) and by district (Figure 3). The number of cases diagnosed by month peaked during November to February and during June to August in North-central region and during March to May and August to November in the North-west region.

Geographically, 86% of the cases resided in 9 (39%) of 23 districts in the study area. The districts with many cases tended to be those nearest to the two hospitals, but there were exceptions. For example, Adjumani, Dokolo, and Pader districts, which are near St. Mary's Hospital, Lacor, had few cases, whereas districts such as Lira and Apac, which are far from St. Mary's Hospital, Lacor, registered many cases.

### Attendance at local health facilities

There were 1012 children at the four HCII facilities in 10 days (Table 2). Most (91%) of the children were found at facilities in North-central region; only 9% were found at facilities in the North-west region. Daily attendance at HCII facilities varied widely from day to day. The average was 40-50 (range, 13-75) children per day at clinics in the North-central region and 2-7 (range, 1-11) at clinics in the North-west region. Attendance did not differ by gender.

**Table 2:** Characteristics of children attending four representative local health facilities in Northern Uganda in May/June 2010

	North-central		North-west		Total	
**Characteristic**	**n**	**%**	**n**	**%**	**N**	**%**

**Sex**						

Male	488	53	39	44	527	52

Female	436	47	49	56	485	48

**Age Group, Years**						

0 - 2	716	79	7	8	723	73

3 - 5	158	17	31	35	189	19

6 - 8	21	2	25	28	46	5

9 - 11	8	1	15	17	23	2

12 - 14	5	1	10	11	15	1

**Reasons for attending facility**						

Malaria	521	55	56	64	577	56

Respiratory tract infection	291	31	12	14	303	29

Urinary tract infection	16	2	0	0	16	2

Gastroenteritis	54	6	5	6	59	6

Skin	36	4	2	2	38	4

Other	22	2	13	15	35	3

Not recorded	2	0.2		0	2	0.2

**Blood test for malaria done**						

Yes	399	77	6	11	405	70

No	122	23	50	89	172	30

**Malaria smear results**						

Positive	197	49	6	100	203	50

Negative	202	51	0	0	202	50

In contrast to BL cases, the average age of children attending health facilities was 2.2 years (SD, 2.8). Furthermore, 73% of children at the local clinics were aged 0 to 2 years; 92% were aged 3-5 years; only 8% of children were aged 8-14 years. Of interest, children at facilities in North-central region were younger than children at facilities in North-west region (1.8 years versus 6.6 years, P < 0.001).

More than half (56%) of children attending the facilities were clinically diagnosed as having malaria and close to one-third (29%) were diagnose as a having respiratory tract infection. However, among 405 children suspected to have malaria who were tested by blood microscopy, malaria was confirmed in only 203 (50%). Confirmed malaria was more frequent in children aged 0-2 years than older children (29.1% versus 20.4%; P = 0.004).

## Discussion

Using historical BL data to guide our selection of controls by age and geography, we found that many children attend local health facilities suggesting that it may be feasible to enrol controls at local health facilities. However, substantial difference in the mean age of BL cases versus the mean age of children found at local health facilities was observed. Specifically, 92% of children at local health facilities were aged 0-5 years. This percentage is 2.4 times higher than the proportion of children aged 0-5 years in the general population in Uganda [22] and 2.6 times higher than children with historical BL in this region [23]. Based on these differences in age pattern, local health facilities may be appropriate sites for recruiting controls for young BL cases, but they may not be appropriate sites for recruiting controls for older BL cases. Theoretically, one could enrol older controls at local health facilities by prolonging the period of enrolment at the facilities, but such a plan requires one to assume that the few older children who are found at local health facilities during the extended period of enrollment are representative of older children in the general population. Because EMBLEM is being conducted in rural areas, we worry that prolongation of enrollment would lead to a "Hawthorne bias" [24,25]. A Hawthorne bias occurs when subjects modify behavior because of actions by the investigator.

Our study also noted that malaria, which is a central exposure under investigation, is highly prevalent in children attending health facilities, especially young children. Given the absence of reliable data on malaria prevalence in children at health facilities versus in the general population, the high prevalence of malaria in children at local health facilities is a concern. Alternative methods of sampling controls were considered. We considered school-based controls, but rejected them because children in Uganda start attending school at age 7 years, thus pre-school children would be excluded. We considered using other cancers as controls, similar to previous case-control studies of BL in Africa [7-9,26], but this idea is not feasible in the EMBLEM study area because other cancers are rare and not well diagnosed. We considered hospital controls, but rejected this idea after reviewing the hospital admissions charts and finding that hospital controls were much younger (mean age, 2 years) than BL cases, and come from villages near the hospital than did children with BL. Finally, we considered neighbourhood controls, as has been used in previous studies [9,26]. We were concern about overmatching and lack of mechanism to verify compliance with selection procedures during implementation.

Given these results, the study design was refined to obtain population-based controls sampled directly from their homes in the village. For practical reasons, the controls will be obtained from representative 100 villages randomly selected from all villages in the study region. The villages were selected after stratifying by proximity to water defined as village having a boundary within or ≥ 500 m of a swamp, river, or lake, and by rural and urban status, defined according to population density from the national census [22]. Stratification was necessary because malaria transmission and BL risk correlated with proximity to water and urban status [27,28]. Children in households selected randomly from a household list are approached and matched according to a pre-specified age and sex eligibility criteria will be invited to participate in EMBLEM as controls.

Our current study does not conclusively evaluate the validity of population versus facility-based controls. Thus, we included a pilot component where both population and facility-based controls will be obtained in 12 villages to examine this issue. Specifically, population pilot controls (PPC) selected from the village (90 per village) will be compared to health centre pilot controls (HCPC) enrolled at the HCII (30 per health facility serving the village). These controls will be compared for distribution of asymptomatic, mild malaria, and malaria-resistance genotypes to determine whether one group is invalid and, if so, to what extent.

To conclude, we compared and contrasted age and sex patterns of historical BL patterns with patterns of children attending local health facilities to gain insights about feasibility and appropriateness of local facilities as site to enrol controls for BL. We noted substantial differences in the age distribution of BL cases versus children at local health facilities leading us to conclude that local health facilities might not be suitable for enrolling controls for older BL cases. To address this concern, the study design was refined to use population-based controls from representative number of villages. A pilot component was included to investigate bias that may be related to using different controls.

## Competing interests

The authors declare that they have no competing interests.

## Authors' contributions

SB conducted field work, reviewed data, analyzed and interpreted data; MM helped collect data and analyzed data; MW interpreted data; MDO supervised field work and interpreted data; TK helped with data collection, analysis, and interpreted data; BE interpreted data; JLZ supervised field work and interpreted data; SJR supervised field work and interpreted data; SMM conceived the idea, designed the study, supervised work, interpreted data. All authors contributed to writing the manuscript and approved the final draft.
